# Is the oral microbiome a source to enhance mucosal immunity against infectious diseases?

**DOI:** 10.1038/s41541-021-00341-4

**Published:** 2021-06-02

**Authors:** Camille Zenobia, Karla-Luise Herpoldt, Marcelo Freire

**Affiliations:** 1 Medical Affairs, Syneos Health, Summit, NJ USA; 2 Department of Biochemistry, University of Washington, Seattle, WA USA; 3 Departments of Genomic Medicine and Infectious Diseases, J. Craig Venter Institute, La Jolla, CA USA; 4 Division of Infectious Diseases and Global Public Health, Department of Medicine, University of California San Diego, La Jolla, CA USA

**Keywords:** Vaccines, Drug discovery, Infectious diseases

## Abstract

Mucosal tissues act as a barrier throughout the oral, nasopharyngeal, lung, and intestinal systems, offering first-line protection against potential pathogens. Conventionally, vaccines are applied parenterally to induce serotype-dependent humoral response but fail to drive adequate mucosal immune protection for viral infections such as influenza, HIV, and coronaviruses. Oral mucosa, however, provides a vast immune repertoire against specific microbial pathogens and yet is shaped by an ever-present microbiome community that has co-evolved with the host over thousands of years. Adjuvants targeting mucosal T-cells abundant in oral tissues can promote soluble-IgA (sIgA)-specific protection to confer increased vaccine efficacy. Th17 cells, for example, are at the center of cell-mediated immunity and evidence demonstrates that protection against heterologous pathogen serotypes is achieved with components from the oral microbiome. At the point of entry where pathogens are first encountered, typically the oral or nasal cavity, the mucosal surfaces are layered with bacterial cohabitants that continually shape the host immune profile. Constituents of the oral microbiome including their lipids, outer membrane vesicles, and specific proteins, have been found to modulate the Th17 response in the oral mucosa, playing important roles in vaccine and adjuvant designs. Currently, there are no approved adjuvants for the induction of Th17 protection, and it is critical that this research is included in the preparedness for the current and future pandemics. Here, we discuss the potential of oral commensals, and molecules derived thereof, to induce Th17 activity and provide safer and more predictable options in adjuvant engineering to prevent emerging infectious diseases.

## Introduction

**Fig. 1:**
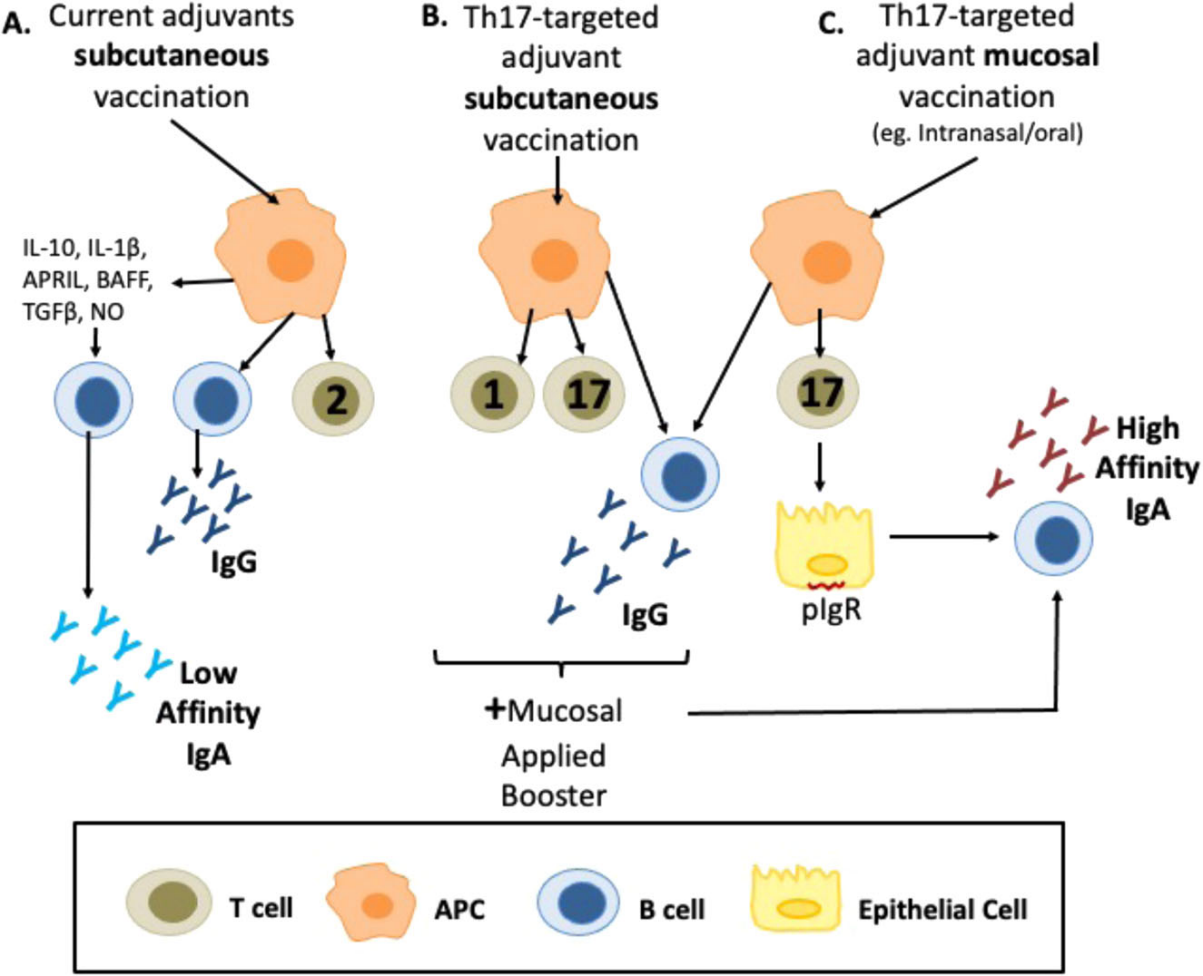
Mechanisms of traditional vaccine versus mucosal induction of cell and serum mediated protection. **A** Current adjuvanted vaccines are administered subcutaneously and induce primarily a Th2 mediated cellular and pronounced IgG antibody response. **B** Experimental adjuvants seeking Th17 protection elicit primarily Th1(1) sometimes accompanied by Th17 (2) cell mediated in addition to the IgG response when applied subcutaneously (3). When combined with a mucosal booster, these experimental adjuvants can offer a robust high affinity IgA response that confers additional mucosal protection (1,2). **C** Experimental adjuvants specific for Th17 can also elicit robust serum IgG when administered via mucosal tissue (4) along with robust Th17 and mucosal IgA with utilization of pIgR (3). Sources^194–198^.

Mucosal barriers throughout the oral, nasopharyngeal, lung, and intestinal systems, offer protection against potential pathogens and exogenous invaders. An abundance of T-helper 17 l (Th17) cells occupy these tissues and mediate serotype-independent immunity and promote mucosal immunoglobulin A (IgA) protection^1–5^. Traditional vaccines are applied parenterally with adjuvants meant to induce a powerful serotype-dependent response which often fail to drive mucosal immune protection, illustrated in Fig. 1. Alum, the current gold-standard in vaccine adjuvants is known to elicit a Th2 response, some pathogens require different cellular immunity. For example, *Bordetella pertussis* is re-emerging as an infectious disease despite having a current vaccine. The current adjuvant employed in the *B. pertussis* vaccine, provided maximum protective immunity requiring Th1 and Th17 responses^6^, not Th2 response. Accordingly, within the last decade, efforts to induce a more productive mucosal response have targeted therapies toward induction of memory Th17 immunity in hopes of gaining broader protection against pathogens that historically have resisted traditional vaccine strategies; Table 1 provides examples of pathogens that naturally elicit Th17 protection but whose vaccines, if available do not. Experiments targeting Th17 have shown mixed results; success on one hand with confirmed induction of Th17 memory and on the other hand evidence that Th17 protection may come with exacerbated pathology upon rechallenge^7,8^. Achieving successful Th17 protection will likely require a new vaccine adjuvant. Currently there are no approved adjuvants for Th17 induction and therefore, the research is littered with experimental molecules which stand to have a long regulatory hurdle. Yet, the host microbiome is uniquely adapted to mucosal surfaces, with an ability to modulate the IL-17 environment, making it a potential source of naturally derived mucosal adjuvants.

**Table 1:** Mucosal pathogens that require Th17-mediated protection.

Pathogen	Natural Infection Cell-mediated Protection	Vaccine Induced Cell-mediated Response	References
*Bordetella pertussis*	Th1 and Th17	Th1 (whole cell), Th2 (acellular)	^180,181^
*Candida albicans*	Th1 and Th17	No vaccine	^182^
*Yersinia pestis*	Th1 and Th17 (Attenuated strain)	No vaccine	^183^
Adenovirus Vector	Th1 and Th17	N/A	^184^
*Mycobacterium tuberculosis*	Th1 and Th17	Th1, Th17, *Th2, *Treg *associated with poor outcomes	^185^
*Helicobactor pylori*	Th17	No vaccine	^4,186^
Influenza virus	Th1 and Th17	Th1	^4,187^
*Pseudomonas aeruginosa*	Th17	No vaccine	^4,188^
*Streptococcus pneumonia*	Th17	No vaccine	^4,189^
*Klebsiella pneumoniae*	Th17	No vaccine	^4,20^
*Aspergillus fumigatus*	Th17	No vaccine	^4,190^
*Blastomyces dermatitidis*	Th17	No vaccine	^4,191^
*C. posodasii*	Th17	No vaccine	^4,191^
*H. Capsulatum*	Th17	No vaccine	^4^
*Herpes Simplex Virus*	Th17	No vaccine	^192^
HIV	Reduced Th17 compartment associated with poor viral control	No vaccine	^193^

List of pathogens that have been shown to elicit natural immune protection with induction of Th17-memory response. Included are vaccines currently available and the T-cell mediated response associated with protection.

**Fig. 2:**
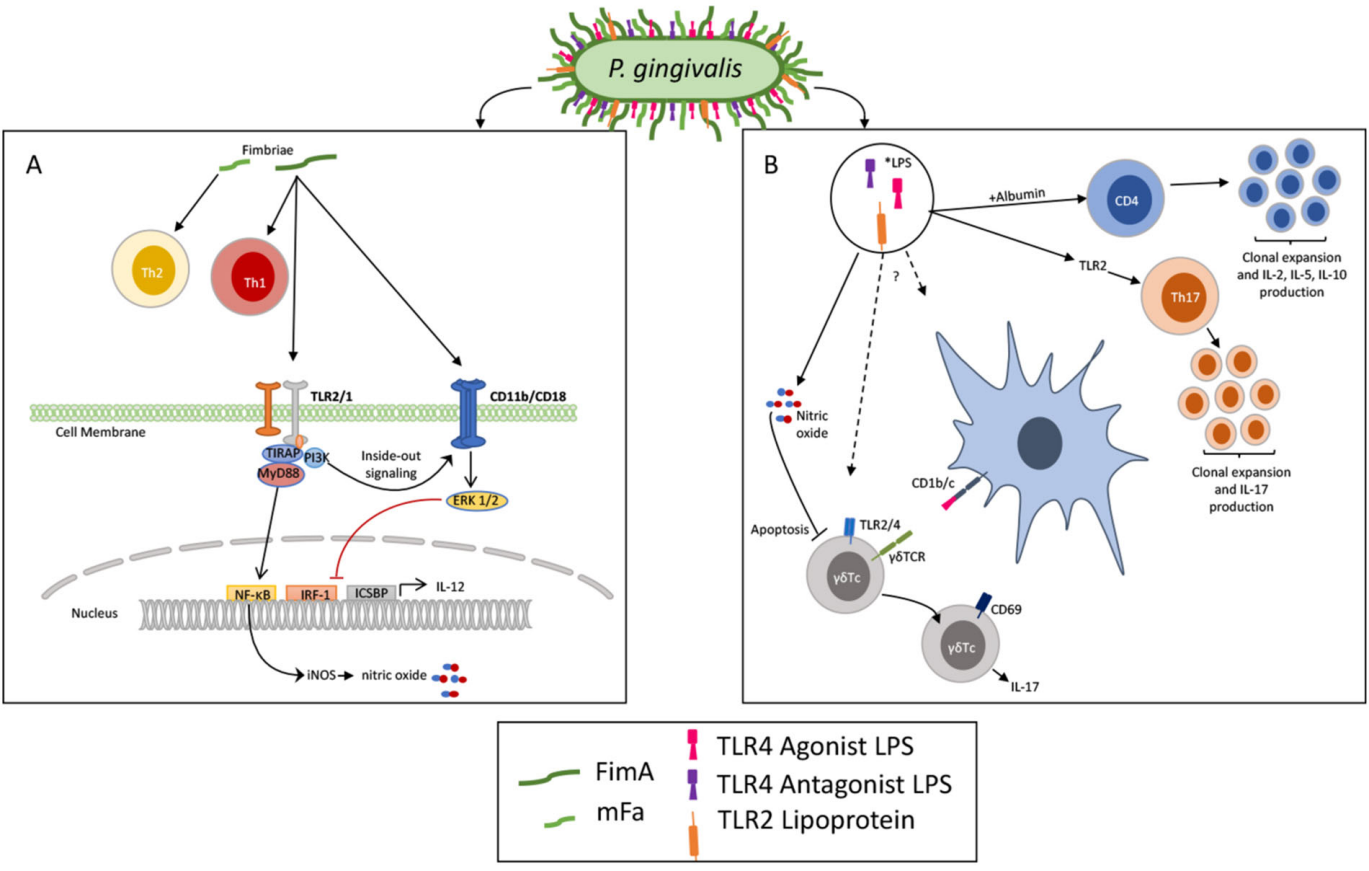
Direct and Indirect antigens derived from P. gingivalis in induction of T-cell functions. In the left panel mFA is shown to induce a Th2 response whereas FimA elicits Th1 activity, it is not known if these effects are direct or indirect but FimA can signal through TLR2 to upregulate NO and CD11b/CD18 integrin expression. FimA also acts as a ligand to the CD11b/CD18 integrin. In the right panel, the products that purify out from the LPS-extraction method are shown; antagonist and agonist LPS (pink and purple) and the lipoprotein from gene product PG1828 (orange). Together, this ‘LPS’ can elicit CD4 T-cell, and Th17 expansion as well as IL-17 from γδTc subsets. Although the signaling pathways for T-cell induction are not yet identified, LPS typically signals through TLR4 but can also be presented (lipid A) by DCs via CD1b or CD1c.

The mucosal tissues are home to constituents of the human microbiome shown to influence the activity of the Th17 response^9–11^ recently reviewed here^12^. Often overlooked in mucosal immunology is the oral cavity where the microbiome has unique and shifting abilities to shape the immune landscape. Th17 cells appear in response to mastication and are then influenced by the microbial milieu^10,13^. Additionally, ɣδT-cell-secreting IL-17 cells that contribute to innate homeostasis functions in the oral tissues, have also been found to be regulated by the oral microbiome^9^. Intriguingly, IL-17 from ɣδT-cells, regulated by microbiota seem critical for induction of Th17 responses in the mouse genital mucosa suggesting the microbiome may help bridge the IL-17 innate immune response to adaptive memory^14^. More recently, a dysbiotic oral microbiome enhanced intestinal pathology in a mouse model of colitis significantly more than the healthy microbiome^3,15^. This study underscores the ability of oral microbes to influence the immunological landscape in the gut where the microbiome has been found to tailor the Th17 response in irritable bowel syndrome^3,15^. Although it is well understood that a dysbiotic oral microbiome can drive IL-17 related gingival pathology, the effects of oral bacteria are less explored^16^. However, there is a great deal of research targeted at understanding *Porphyromonas gingivalis* (*P. gingivalis*), an inflammophillic constituent of the oral microbiome most notable for its low abundance contribution to periodontal disease^17^. *P. gingivalis* lipopolysaccharide (LPS) has a unique capacity to induce CD4+ T-cell activity in an ovalbumin-challenged mouse model^18^ found prior to the discovery of the Th17 cell type. Since then, research has shown that *P. gingivalis* LPS induces Th17 differentiation via Toll Like Receptor (TLR)-2 activity (Fig. 2)^19^. This unique activity from *P. gingivalis* LPS may provide a natural adjuvant quality to the oral microbiome not previously recognized. As research into the host microbiome in the context of IL-17 mucosal regulation and Th17 memory subsets expands, additional preferred induction pathways and additional natural adjuvants will likely be identified.

In the last ten years have there been efforts to target a memory-Th17 response in the context of vaccine therapy. Th17 protection is particularly attractive because these memory T-cells have been shown to protect against heterologous pathogens in an antibody-independent manner^20^. This is a particularly pertinent issue as coronavirus vaccine candidates race through clinical trials; the potential protection offered by a Th17 response that may protect against other coronavirus strains/mutations is a very attractive proposition. Protection from vaccine-induced memory Th17 cell response has been described for *Candida albicans, Streptococcus pneumoniae, Staphylococcus aureus, Pseudomonas aeruginosa* and *Mycobacterium tuberculosis*^21–24^. While the Th17-targeted vaccine protection from fungal and bacterial infections appear successful, the efforts to design similar vaccines for protection from viral infections have been fraught with complications. Specifically, studies investigating Th17 memory response for lasting influenza protection have been successful in producing effective memory response but have shown exacerbation of pathology upon challenge^7,8^. The route and site of inoculation appear to play a key role in effective induction of Th17 response, indicating that induction of mucosal memory might be more complex than initially thought^25^. Currently there are efforts to improve our understanding of the numerous pathways of mucosal induction of Th17 and to identify successful adjuvants that will ensure safe, but effective vaccine responses. In an attempt to provide some insight into these issues, we review the role of the oral microbiome, specifically in terms of Th17-immune induction and discuss its potential role in harnessing natural adjuvants that might be utilized for future vaccine therapies.

## Mucosal IL-17-producing T-cells

Mucosal tissue is characterized by epithelial cells, connective tissue and resident myeloid and lymphoid cell types. Within these tissues reside IL-17-producing lymphocytes, which can further be divided into ɑβ and ɣδ T-cells (ɑβTc and ɣδTc). The ɣδTc have been described as an innate-type of T-cell that are unrestricted by major histocompatibility complex (MHC) class molecules, that appear required for macrophage recruitment and differentiation upon bacterial challenge^26–29^, and can, upon direct stimulation, produce the antibacterial products granulysin and defensin to provide barrier protection^26,27^. The IL-17+ɑβTc, more commonly known as Th17 mediate a heterologous mucosal immunity that is independent of antibody response^20,30^. The ɣδTc have been shown to shape the T-cell repertoire in the mucosal tissues and contribute to induction of Th17 memory subsets^14,31,32^. Although both Th17 and ɣδTc^33^ can develop memory subsets that respond rapidly to challenges specific to mucosal protection, Th17 immunity is linked to the production of a protective IgA response^5^ making the Th17 cells attractive targets for vaccine design.

The ɣδTc are primarily described as innate responders in the mucosal tissues, which maintain epithelial cell integrity and are also capable of bridging the innate to adaptive immune response^34–36^. Similar to the classical T-helper cell, the ɣδTc can develop into distinct subtypes and specific effector profiles to produce an array of chemokines and cytokines including INF-ɣ, TNF-ɑ, IL-17, IL-21 and Il-22^37^. When stimulated, the naive ɣδTc will produce IL-17. However, if antigen-experienced, the ɣδTc will secrete INF-ɣ^38^. The production of different ɣδTc subsets during RAG-mediated recombination of V(D)J in the thymus gives rise to several subtypes most notably the Vγ9Vδ2+ ɣδTc population primarily seen in human peripheral blood and the Vδ1+ which are found in the epithelium^36,37,39^. The ɣδTc are capable of sensing and responding to self-antigens as well as bacterial antigens both peptide and non-peptide, such as the lipid-A moiety of LPS^39,40^. ɣδTc activity can elicit a protective Th17 or autoimmune response^14,32,41^, the difference being likely the result of local cytokine milieu reviewed here^42^. The authors postulate that induction of INF-ɣ+Th17 is protective in a vaccine model while GM-CSF may confer autoimmune pathology. The stimulation of ɣδTc by bacterial lipids or fungal β-glucan through TLR2 or Dectin-1 respectively produces rapid induction of IL-17^43^. IL-17 and INF-ɣ production by ɣδTc can also be induced by exposure to IL-1β and IL-23 and contribute to a Th17 response^37^. Although, the cytokine milieu plays an important role in activation of the ɣδTc, so, too, is the location of the ɣδTc. The subset of ɣδTc found in the dermis seems important for induction of a protective Th17 response. Complete Freund’s Agent (CFA) adjuvant was found to stimulate a dermal subset of ɣδTc to induce a Th17 profile that included IFN-ɣ+ɑβ-Tc and TNF-ɑ+ɑβ-Tc subsets; when the ɣδTc was specifically blocked via Vɣ, the ɑβTc profile shifted to ɑIL-6+βTc^32^. The circulating ɣδTc did not appear to be effective in this model of Th17 differentiation indicating a unique role for the tissue ɣδTc subset for directing the mucosal IL-17-mediated immune response. The ɣδTc have also been implicated for their role in shaping the natural antibody repertoire that participate in host defense, autophagy, tissue remodeling and immune regulation^44^. Through a series of ɣ- or δ- subtype T-cell-deficient mouse strains, the authors discovered that the peripheral ɣδTc subset with cooperation from ɑβTc shape the natural antibody response in the non-immunized state. With the understanding that the ɣδTc are influenced by the shifting constituents of the microbiome whose presence can contribute to changes in the cytokine milieu and influence specific adaptive immune memory, a deeper examination of the bacterial influence during both treatment and outcome is warranted.

Heterologous or universal protection is perhaps the “Holy Grail” for lifetime vaccine protection against seasonal viral challenges that result in loss of life rather than the current annual vaccine requirement. Heterologous protection has been demonstrated with bacterial vaccines where Pneumococcal vaccines have been found to protect against more than 90 pneumococcal serotypes. Observations like this one have led to the development of novel methods that leverage the homology between species. *Streptococcus mitis*, for example, an oral commensal^45^ that shares immunogenic characteristics with *S. pneumoniae* was recently utilized as a vaccine candidate. Shekhar et al. evaluated the potential of *S. mitis* and its mutant expressing the pneumococcal capsule type 4 (*S. mitis* TIGR4cps) to induce protection against *S. pneumoniae* lung infection in mice. Their findings demonstrated that intranasal vaccination with *S. mitis* provided protection against two *S. pneumoniae* strains in a serotype-independent fashion, which was associated with enhanced antibody and cell-mediated responses, including increased Th17 immunity^46^. Recent advances in vaccine research have illuminated a role for the induction of Th17+ and Th1+ to confer vaccine protection against multiple influenza strains which indicate that a universal vaccine is possible^27,47–49^. IL-17 plays an integral role in immune protection following viral infection. Th17 differentiation can be induced by IL-17 from ɣδTc whereas IL-17 from Th17 has been shown essential for mounting an effective Th1 response against viral infection^50^. Th17 cells contribute to a cell-mediated, clade-specific, serotype-independent immunity with the additional ability to induce high-affinity mucosal IgA, heterologous protection and memory Th17 subsets effective for vaccine-induced protection^5,20,51,52^.

In order to stimulate an effective immune response to viral pathogens, much recent vaccine work has centered around different adjuvant systems targeting Th17. Intriguingly, evidence is mounting in support of two-component adjuvant systems for induction of Th17-immune protection to increase efficacy and improve safety. One adjuvant named CAF01 combines a mycobacterial fusion protein with a cationic liposome that function together to promote antigen presentation cell (APC) uptake and signaling through Dectin-1^53^. Although the signaling pathways utilized by CAF01 are not fully understood, they have been shown to signal in part through the MyD88 pathway^54^ with likely interactions that include integrin and TRIF/Inflammasome engagement due to the cationic liposome component^55^. In another study, the adjuvant consisted of a lipidated TLR7/8 agonist and a synthetic TLR4 agonist, that when applied together elicited a more potent Th1+ Th17+ response than when adjuvants were applied individually^47^. Another study evaluated a nanoemulsion combination of dioacetyl dimethyl ammonium chloride (DODAC) named NE01 as an adjuvant system, and found it to be effective only when a booster dose was added^49^. A subsequent study found that by substituting cetytlpyridinium chloride (NE02) for DODAC and adding a TLR9 agonist, the vaccine challenge produced a very rapid and specific humoral response that conferred reduced toxicity with improved health outcomes^56^. NE02 was previously characterized for its ability to induce IL-17, although the signaling pathway was not identified^57^. This last study underscores the possibility that a second component for induction of a Th17 vaccine response may be required to improve vaccine efficacy *and* safety. To further illustrate this, in a study of influenza vaccine in a mouse model, the adjuvant CRX-601 (a TLR4 agonist) was used to stimulate a Th17 response; however, results came with a detrimental neutrophilic lung response^8^. A subsequent study found that by combining CRX-601 with chitosan in liposomes, the safety and vaccine efficacy was improved^58^. Chitosan is known for utilizing many cellular pathways such as TLR4, Dectin-1, and CR3, reviewed here^59^. Together, these studies illuminate a distinct role for engagement of multiple pattern recognition receptors (PRR) for the induction of a safe, effective and potentially heterologous mucosal protection driven by IL-17-induced cell-mediated immunity. This is compelling given the fact that the human microbiome is continually modulating PRRs that contribute to an IL-17-response and may likely confer some adjuvant quality, especially for therapies that utilize sublingual, buccal or intranasal inoculation where the bacteria colonize.

Indeed, the host-microbiome has been shown to function as an adjuvant during vaccine treatment. A study to evaluate the nasal microbiome and efficacy of IgA-seroconversion during the challenge of a live-attenuated, seasonal influenza vaccine found that carriage of several different commensal species but perhaps most significantly *Lactobacillus helveticus* and *Bacteroides ovatus*, could increase IgA specificity to the vaccine^60^. Although it is understood that the success of a mucosal vaccine response will involve Th17 to gain IgA protection, the T-cell repertoire was not evaluated in this study so it is unknown how the commensals may have contributed to T-cell activation during seroconversion. The commensal (and sometimes pathogenic) *Staphylococcus aureu*s can stimulate recruitment of CD11b+ CCR2+ monocytes that mature into M2 macrophages and dampen influenza-mediated acute lung injury^61^. How this outcome may affect the T-cell repertoire is unknown yet: a study of *Staphylococcus aureu*s in persistent carriage compared the IL-17, Th17 and T-regulatory (Treg) response to non-carriage controls^62^. It was discovered that carriage came with a diminishment of both IL-17 and Th17, with no change in IFN-ɣ. Together, these studies illuminate the potential of *Staphylococcus aureu*s in protection against severe influenza infection potentially arising from a reduced but not ablated Th17 response. The impact of the host-microbiome relationship cannot be understated. Evidence is building around a role for the oral microbiome in health outcomes in which a healthy microbiome and coordinated actions with the immune system can maintain homeostasis. While dysbiotic inflammatory networks^63^ derived from microbiome induction trigger a cascade of events that lead to oral diseases such as caries^64^, periodontal diseases^17^, oral cancer^65^. In addition, due to this dysbiotic relationship oral-systemic diseases^66^ have also been implicated including diabetes^67,68^, cardiovascular disease^69^, diabetes, and IBS^3,15,70^. Further, the host-microbiome (both oral and gut) has been implicated in nosocomial and ventilator-associated pneumonia where a dysbiotic microbiome can increase the abundance of respiratory pathogens^71–73^ and contribute to superinfections^74^. The superinfection in this case was driven by alterations to the host microbiome from influenza infection. Alterations to the host microbiome have been documented during infection with both influenza and coronavirus^56,75,76^, but the understanding of how microbial members may impart immune protection or contribute to a successful/detrimental vaccine is lacking. A fairly recent review of the microbiome impact on vaccine response examines the issue of protective versus detrimental microbiota in vaccine efficacy^77^. The authors discuss the health outcome disparity of vaccine response between countries with a focus on factors that influence host-microbial diversity such as diet and gastrointestinal infections. The overwhelming take-home message is that a dysbiotic microbiome can negatively affect vaccine efficacy since microbial diversity and TLR-engagement drive the T-cell activity required for successful therapeutic outcomes. Although these studies provide evidence that the host microbiome can contribute adjuvant activity, the specific pathways for successful antiviral protection remain undefined.

Some significant progress in understanding antiviral T-cell response has emerged from the rapid study of SARS-CoV-2 infection. First, several studies published recently with regard to SARS-CoV-2 indicated that healthy, seronegative donors may have heterologous protection from the current coronavirus strain due to previous coronavirus infections^78–80^. In Braun et al., the T-cells obtained from both recovered (83%) and healthy donors (34%) showed reactivity from the SARS-CoV-2 spike glycoprotein^78,79^. In depth epitope mapping and characterization provided evidence of cross-reactive immunity to SARS-CoV-2 from prior coronavirus exposure. These data are compelling evidence to suggest a universal coronavirus vaccine might be possible. The cited studies above indicated that both CD4+ (likely Th1 dominant) and CD8+ T-cells were capable of reactivity but no further characterization was provided. A recent study of T-cell characterization for patients with COVID-19 describes a dominant Th1 repertoire with additional but smaller subsets of Th2 and Th17^80,81^. It remains unclear what type of T-cell repertoire would be found in an asymptomatic person or the patient successfully controlling a SARS-CoV-2 infection which is potentially of greater importance for informing vaccine targeting strategy. There is emerging discussion about IL-17 and Th17 as detrimental in coronavirus immunopathology and concerns around vaccination-induced immune enhancement reviewed here^80–82^. However, an epidemiological study of patients on current IL-17 inhibitors indicated that these patients were at higher risk for respiratory diseases compared to controls^83^. Given that the dominant T-cell response in SARS-CoV-2 infection seems to be Th1 with a subset of Th17 and, combined with the understanding that the inhibition of IL-17 may lead to increased viral susceptibility, it is attractive to consider that the Th17 may be playing a supportive role for induction of a potent Th1 response. Indeed, there is some additional evidence that the addition of IFN-ɣ can reduce IL-17 mediated pathology^84^. This is the mechanism by which adjuvant activity is directed towards a Th17 response that confers Th1 memory might improve vaccine design for a safe and effective mucosal immunity with potential heterologous protection, a particularly attractive notion given the current drive for a successful SARS-CoV-2 vaccine. Additionally, it might be important to evaluate the host-microbial influence on disease severity and therapeutic outcome especially if the therapeutic application is at the mucosal interface.

## Immune landscape of the oral mucosa

Humans are host to a multitude of microorganisms that co-develop from birth, and are dependent on the host genome, nutrition, and lifestyle^85^. The oral microbes have evolved with host tissues over thousands of years and are specifically adapted to the mucosal tissues^86^. The human oral cavity is home to one of the most complex microbial ecosystems within the body. Estimates suggest there are more than 750 bacterial species in the human oral cavity, many of which have been implicated in local and systemic diseases. The sequential organization of the oral microbiome is complex, niche-dependent, and distinct in health and disease. Salivary mucins and proline-rich proteins help initiate bacterial colonization and biofilm formation^87^, but also act to aggregate and clear the bacteria from oral surfaces^88^. Although biomechanical and biochemical cues control initial adhesion of microbial biofilms on hard and soft tissue surfaces, multiple signaling molecules facilitate, such as cytokines, protect microbial colonization as well as participate in the development and maintenance of a healthy immunity at the mucosal border. For example, the gingival crevice has been shown to express an interleukin 8 (IL-8) gradient in the junctional epithelium which serves to recruit neutrophils into the tooth pocket where bacteria naturally accumulate^89^. Once in the gingival sulcus/pocket, neutrophils form a barrier between the junctional epithelium and the subgingival biofilm. The neutrophil-wall prevents the apical migration of the bacteria deeper into tissues, which is essential to maintain periodontal homeostasis^90^. Thus, preventing microbial access to connective tissue, major blood vessels and migration to systemic sites.

Out of all immune cells present in the oral mucosa and gingival tissue, neutrophils constitute 95% of total leukocytes present in oral tissues. Transmigration of neutrophils is continuous through gingival and buccal mucosal tissues, with 30,000 neutrophils per minute passing through the highly permeable epithelium, namely the junctional epithelium^91^. The neutrophil is the main immune cell that coordinates anti-bacterial responses in the gingival tissues primarily by utilizing proteases, defensins, cytokines, phagocytosis and neutrophil-extracellular-nets (NET); its function is intimately tied to IL-17 production^92,93^. Although few in number, other important immune cells reside in the gingival tissues: these include resident T- and B-cells (rare in healthy tissues), innate lymphoid cells (ILCs), macrophages, and dendritic cells (DCs). CD4^+^ and CD8^+^ T-cells produce specific types of cytokines, including canonical types 1 and 17. Additionally, novel patterns of B-cells and plasmacytes have been identified in gingival tissues^94,95^. T regulatory cells (Tregs) also reside in the gingival tissue and provide immunological tolerance^94,96,97^. M2 macrophages and DCs are found in small numbers in the healthy gingiva, with a subset of CD103^+^ dendritic cells that provide yet another level of barrier function, while M1 macrophages increase during inflammatory insult^98^.

The neutrophil interplay with the macrophage is a central component to mucosal tissue homeostasis. The tissue macrophage produces signals, including IL-23 which is followed by an induction of IL-17 from nearby T-cells, shown to regulate neutrophil granulopoiesis^92^. When sequestered into the tissue by damage or infection, the otherwise short-lived neutrophil is activated by microbes and/or local chemokines (e.g., IL-8), thereby inhibiting apoptosis programs, which allows the neutrophil to coordinate an appropriate cellular response^99,100^. Once phagocytosis of the infectious particle occurs, the neutrophil typically undergoes apoptosis^101^. The apoptotic neutrophil then signals to the monocyte, triggering the second wave of host-mediated immune responses^102^. Endocytosis of the apoptotic neutrophil by the monocyte or macrophage downregulates IL-23 and IL-17 production^92^, reduces inflammation and initiates pathways of wound healing^102,103^. If neutrophils are unable to move into tissues to coordinate cleanup as in the case of Leukocyte Adhesion Deficiency (LAD)-1 or if neutrophil apoptosis is delayed, the IL-23 and IL-17 cytokines persist and contribute to exacerbated tissue inflammation^104,105^. The IL-23-IL-17 tissue program has been shown to be essential for tissue homeostasis in gingival mucosa and, if disrupted genetically by bacterial manipulation or hormonal changes, IL-17 can contribute to inflammatory pathology^104,106,107^.

Germ-free animals have been utilized to uncouple the gingival tissue program from microbial influence. These studies have shown that as the gingival tissue develops early in life, microbial independent tissue sequestration drives an influx of IL-17 secreting-γδT and chemokine C-X-C-ligand (CXCL)-1-dependant neutrophil recruitment to basal and junctional epithelium while the acquisition of the oral microbiome alters the γδT subtype and coordinates additional neutrophil recruitment via CXCL2^9,108^. Microbial-independent mastication forces drive Th17 cells into the gingival tissues while a distinct dysbiotic microbial community can initiate Th17-dependent oral pathology^10,13^. During the aging process, increases in the number of Th17 are found with apparent simultaneous decreases to the γδT in the gingival tissues^9,10,13,108^. These IL-17-producing T-cell profile alterations coincide with significant bacterial burden, IL-17-related bone loss pathology^109^ marked by a distinct and significant rise in neutrophil activity. Combined, these studies illustrate a very unique tissue program, which underscores a role for γδT-IL-17 in homeostasis that is heavily influenced by age and the bacterial community.

The neutrophil has been shown to regulate ɣδTc. Two neutrophil-derived products, neutrophil elastase (NE) and reactive oxygen species (ROS) have been found to exert opposing effects on ɣδTc; activating the T-cells with NE and inhibiting with ROS^110^. Both of these factors have been found in the gingival crevice and shown to be stimulated by oral bacteria^111,112^. When NE is specifically inhibited during sublingual immunization, significant increases are found for Th1, Th2, Th17, and for antigen-specific IgG and mucosal IgA when compared to controls^113^. This study underscores the impact of the neutrophil in relation to T-cell activity and B-cell class switch. A prior study by the same laboratory found neutrophils inhibited the B-cell activity mainly through IKKβ. When the IKKβ signal was depleted, IL-17RA+ B-cells increased significantly^114^, which is compelling given that IL-17A has been found to trigger class switch via Th17 helper cell activity^115^. Th17 cells are required for induction of a long-lasting protective IgA response which is an important mediator for the first line of defense in mucosal tissues^5^. Further, a comparison between sublingual and intramuscular influenza vaccine application found that sublingual routes elicited systemic immunity similar to that from intramuscular routes, but only the sublingual application leads to a protective Th17 with mucosal IgA protection^116^.

The interaction between γδTc and neutrophils seems intimately tied. An examination of γδTc found they were capable of inducing antigen-presenting cell (APC)-functions in the neutrophil^117,118^. After exposure (and phagocytosis) to bacterial metabolites, the neutrophils differentiated into APC upon co-culture with the γδTc. Once activated, the neutrophils could present antigens to both CD4+ and CD8+ T-cells. In this scenario, the neutrophils had the ability to flexibly activate either CD4 or CD8 T-cells. To substantiate the finding, study participants with sepsis were shown to have highly activated γδTc, APC-like neutrophils, and activated CD8+ T-cells^117,118^. Neutrophils have been shown to either inhibit or activate T-cell mediated functions depending on cellular responses to the microenvironment. Since neutrophil activity in the mucosal tissue is directly tied to and influenced by bacterial products as well as IL-17 regulation, any vaccines that require the induction of Th17 for protective response may need to be designed around or informed by neutrophil activity.

## Oral microbiome: naturally derived adjuvants

Both the γδTc and neutrophils are essential for maintenance of the mucosal border where the host-microbiome community resides and can respond accordingly through TLRs and complement receptors. The γδTc is also capable of sensing phospho-antigens like LPS-derived Lipid A via γδ-TCR cross-presentation of CD1b or CD1c on APCs or directly through TLR2^119,120^. Much of the interaction between the γδTc and neutrophil is to coordinate the management of a symbiotic relationship with the microbial community, modulating between tolerance and removal^121,122^. It is here where the adjuvant quality of the oral microbiome may impart therapeutic IL-17 activity. Indeed, the IL-17 program is intimately tied to microbial activity with γδTc providing an IL-17 environment that supports homeostasis^9^ while the neutrophil is often associated with IL-17 pathology^16,123^.

Bacteria-derived proteins, polysaccharides and lipids have been utilized as adjuvants to induce specific immune responses and tailor antibody specificity and have been reviewed extensively^124,125^. TLR ligands have long been examined for their adjuvant activity and are currently under investigation for therapies in allergy, cancer, vaccine and autoimmune dysfunction^126–129^. Monophosphoryl lipid A (MPL) is one example of an FDA-approved Toll-like receptor 4 (TLR4) adjuvant. The lipid A structure is derived from the LPS of *Salmonella minnesota* and is currently used in the human papillomavirus vaccine, Cervarix Ⓡ. The lipid A structure of MPL shares some similar features to that of the oral microbe *P. gingivalis*. This oral derived adjuvant is capable of activating cell receptors such as TLR4^130^. The vaccine is now use for HPV-16 and 18 associated cervical cancers^131^. *P. gingivalis* has heterogeneous TLR4 activity found to be caused by the ability to shift its LPS structure, specifically the lipid-A moiety, to that of TLR4 antagonist or agonist^132,133^. *P. gingivalis* can alter its lipid-A structure during changes to environmental conditions such as levels of hemin or temperature, both important features of inflammation activation^134,135^. Two well-characterized lipid-A moieties of the *P. gingivalis* LPS molecule have been shown to exert opposing effects on neutrophil recruitment and are likely the culprits of *P. gingivalis*-induced “chemokine paralysis”: *P. gingivalis* has been found to disrupt epithelial cell expression of interleukin 8 (IL-8), an important chemokine for neutrophil recruitment into gingival tissues^89^. The tetra-acylated structure, characterized as a potent TLR4 antagonist, can reduce recruitment of neutrophils by blocking IL-8 chemokine production in gingival tissues while the penta-acylated structure is an agonist to TLR4 that increases IL-8 production and subsequent neutrophil traffic^89,134,136–139^. LPS activity in the gingival tissues has been implicated in periodontal disease. The LPS activity from the oral bacteria seems to play a role in the progression to periodontal disease, moving from TLR4 antagonist activity in healthy sites towards agonist activity in sites with active disease^140^ and may take on different adjuvant activity in the context of vaccine therapeutics.

The oral microbiome has been implicated in induction of IL-17-related pathology. However, the role of the microbiome also seems to be a factor in γδTc IL-17 homeostasis^9,10^. A dysbiotic oral microbiome has been shown to trigger Th17 activity which resulted in IL-17 associated pathology, whereas ablation of γδTc gave rise to alterations to the microbiome that resulted in IL-17 inflammation and gingival pathology. Although these studies implicate the microbiome in inflammatory pathology, an IL-17-related homeostasis also exists, the difference likely culminating around the activity of the oral neutrophil. In an elegant series of experiments using integrin and developmental endothelial locus-1 (DEL-1)-deficient mice, it was recently shown that apoptotic neutrophil efferocytosis is essential for regulation of IL-17 homeostasis and disruption to this function can result in periodontal disease and dysbiosis^104,141^. During oral dysbiosis, an outgrowth of anaerobic bacteria is described which are often referred to as perio-pathogens. However, these bacteria are also present in healthy tissues albeit at much lower concentrations^142,143^. Many of these ‘pathobionts’ have been studied in terms of their ability to cause tissue destruction. Yet, co-evolutionarily they may contribute some benefit when in low abundance. In support of this hypothesis, a recent study illuminated a role for LPS derived from the oral microbiome and released into gingival tissue that was specifically responsible for the ecological maintenance and balance of the mesenchymal stem cells that reside in the mucosal tissues^144^. In this scenario, the anaerobic bacteria are providing a benefit to host tissues. *P. gingivalis*, a member of the anaerobic community has been shown to modulate IL-17 activity via LPS, fimbriae and the proteases termed ‘gingipains’^145–148^, and induce IL-17 from both Th17 and γδTc^149^. Together, these data provide evidence that *P. gingivalis* has the ability to impact the IL-17 program in multiple ways and may be found to participate in IL-17 homeostasis under normal, healthy conditions.

Although there are many oral microbes that appear to contribute to IL-17 activity in the oral mucosa, *P. gingivalis* and its microbial products are perhaps the most studied in terms of oral mucosal IL-17, T-cell activation, and neutrophil manipulation, reviewed here^123,150^. Both the *P. gingivalis* LPS and gingipain proteases have been implicated in IL-17 and Th17 response^146,147,151^ whereas the fimbrial proteins have been shown to activate a Th1 response^152^ despite the ability to block IL-12^153^ required for Th1 activation. IL-12 has also been found to expand Th17^154^ in which case, the fimbrial proteins may be controlling the amount of Th17 expansion. The focus of potential adjuvant qualities of *P. gingivalis* will remain on LPS and fimbrial proteins due to the association between gingipains and the development of autoimmunity, cardiovascular disease and diabetes^155,156^. The LPS and fimbrial proteins have been primarily described as TLR2 agonists and while true, each pathogen-associated molecular pattern (PAMP) has additional signaling capacity that lends further immune modulatory activities.

### *P. gingivalis* LPS

Depending on the cytokine milieu, *P. gingivalis* derived LPS can contribute to a multitude of host-immune responses that culminate in a specific T-cell response. The purified LPS, described above as having heterogeneous lipid A structures that can modulate TLR4 receptors, and also contains a lipoprotein contaminant encoded by gene product PG1828 which has been evaluated as the main TLR2 agonist^157^. *P. gingivalis* LPS has been shown to expand CD4+ T-cells^18^ and induce Th17 activity in a TLR2-dependent manner^19^. During a footpad challenge with ovalbumin, *P. gingivalis* LPS significantly enhanced antigen specific CD4+ T-cells that released IL-10, IL-2 and IL-5 upon stimulation^18^. IL-2 has been found to modulate tissue-specific γδTc to favor short-term effector activity and preserve survival and plasticity^158^. Further, *P. gingivalis* LPS has been shown to induce CD69 on the γδTc^159^, a molecule required for tissue retention and immune modulation^160^. Repeated exposure of *P. gingivalis* LPS tolerized monocytes which then significantly increased ROS production in neutrophils and inhibited neutrophil chemotaxis^161^. While ROS has been found to inhibit γδTc activity^110^, nitric oxide (NO) has been shown to protect γδTc from apoptosis^162^. *P. gingivalis* LPS can elicit the production of NO in vivo^163^ with a subsequent study showing the involvement of TLR9^164^. Notably, NO from macrophages has been shown to confer γδTc protection in mucosal candidiasis^165^. Although it seems plausible that *P. gingivalis* LPS is likely involved in γδTc activity it is unclear whether the effect is direct or indirect. γδTc can recognize lipid A from LPS via dendritic cell presentation^40,164^ or directly through TLR2^120^ but it is unknown how the heterogeneous lipid A structures of *P. gingivalis* LPS might alter γδTc activity. It should also be noted that *P. gingivalis* LPS can induce Natural Killer T-cell (NK) activity in vivo with less cytotoxicity than *Escherichia coli* LPS^166^. This activity was not shown to be either direct or indirect but it is interesting that the γδTc has been shown to induce NK activity^166,167^ and may be involved during *P. gingivalis* LPS induction due to its ability to sense lipid A. Together, these studies illustrate the capacity of *P. gingivalis* LPS to modulate the T-cell repertoire and drive an antigen-specific response.

### *P. gingivalis* fimbriae

*P. gingivalis* fimbriae are expressed as two distinct proteins, the long, major (FimA) and short, minor (Mfa) fimbriae. Both are required for attachment during colonization^168^ and can differentially modulate T-cell responses. The FimA and accessory proteins (FimCDE) are considered the main colonization factors^169^ and engage TLR2 in combination with CXCR4 or the complement receptors, C3R or C5aR to modulate integrin function and elicit IL-1ɑ, IL-1β, TNFd, IL-6 cytokines, reviewed here^169,170^. Integrin function is intimately tied to T-cell activity required for T-cell translocation from vasculature to periphery, chemotaxis through tissues, and APC-T-cell signaling synapse for initiation of adaptive immune response. The minor and major fimbriae appear to direct differential T-cell activity. In a series of fimbriae-deficient bacterial strains, the minor fimbriae were attributed to a Th2 response whereas the major fimbriae induced Th1 response^171^. The fimbriae activate TLR2 which induces the high-affinity conformation of CD11b/CD18 (aka CR3, MAC-1). The fimbriae can then function as a ligand for CD11b/CD18-blocking integrin activity which includes IL-12 down regulation^172^. CD11b expression on APCs is required for induction of peripheral oral tolerance and suppression of Th17 differentiation^173^. *P. gingivalis* fimbriae may be capable of breaking oral tolerance to promote inflammatory response that can mount an efficient and specific immune response with coordinating antigens. The fimbriae of *P. gingivalis* can direct specific T-cell activation with additional potential to break tolerance. This would likely improve mucosal adjuvant qualities and enhance specificity for therapeutic design, depending on the antigen and protection requirements.

The PAMPs of *P. gingivalis* are perhaps the most studied of all the human oral bacteria and provide a unique opportunity to evaluate host immune modulation during vaccine response. The full list of potential functions is far from adequately investigated. Although the specific PAMPs are not yet identified, *P. gingivalis* has also been shown to induce both Nod-Like Receptors (NOD)-1 and -2^174,175^ as well as TLR7^176^. The NLRs are involved in T-cell activation and when paired with TLRs can be utilized to activate both Th1 and Th17 simultaneously^177^. TLR7 suppresses the Th17 autoimmune response^178^. While the mechanisms at play here require further investigation of specific PAMP activity, the ability of *P. gingivalis* to exert a seemingly unlimited number of options for modulating the immune response is enticing. Certainly, there is an opportunity to utilize specific combinations of both *P. gingivalis* LPS and fimbriae since each contains components with unique capabilities to induce different types of immune response qualities that lend a highly specific adjuvant quality to a given vaccine formulation.

Currently it is unknown whether direct induction of Th17 or γδTc-induced Th17 memory is more beneficial for antiretroviral vaccine response. However, there is evidence that the use of adjuvants that stimulate multiple PRRs can confer higher specificity and increase the safety profile during a Th17-based vaccine response. The range of PRRs that are utilized by *P. gingivalis* PAMPs present an opportunity to coordinate a very specific T-cell response and may be utilized to tailor more effective vaccine therapies. Since *P. gingivalis* is a co-evolutionary member of the host oral microbiome, its PAMPs are likely to have a relatively safe immune profile. The unique pathways of IL-17 regulation during homeostasis in the oral cavity are intimately tied to the oral microbiome. Accordingly, the evaluation of *P. gingivalis* PAMPs, and other oral microbiome derived constituents, in vaccine immune response may very well lead to a new understanding of the natural adjuvanticity of the host-microbiome.

## Conclusions

The diversity of the microbiome has been implicated as a driver for differences in vaccine efficacy across geographical regions. In this vein, the use of probiotics has been investigated as a method to improve vaccine response with some successes. In a meta-analysis of probiotic-use in vaccine clinical study, the authors found that while there was significant variability, the probiotic effect was most notable for orally applied vaccines^120,179^. Some of the studies that were highlighted as successful showcased outcomes that included increased IgA responses and elicited cell-mediated and humoral immunity. It is not known whether the successes of the probiotic applications were due to direct effect of the probiotic or the specific activity of the host-microbiome. Together, these studies underscore the potential of the host-bacterial constituents as a powerful source of adjuvant-activity that could be harnessed for immune-modulation of the vaccine response and may offer a path around the steep regulatory hurdle that most adjuvants incur during development. The oral microbiome is a rich source of potential with regard to adjuvant activity which is convenient since the oral cavity is also the ideal site for vaccine inoculation. Investigation into the microbial products, metabolites responsible for coordinating specific immune protection will likely be a fruitful endeavor for identifying adjuvants, and will also provide a better understanding of how immunity is shaped by our microbiome.

## Data Availability

There were no datasets generated and/or analyzed during the current study.
